# Comprehensive analysis of the association between *UBAC2* polymorphisms and Behçet’s disease in a Japanese population

**DOI:** 10.1038/s41598-017-00877-3

**Published:** 2017-04-07

**Authors:** Kyoko Yamazoe, Akira Meguro, Masaki Takeuchi, Etsuko Shibuya, Shigeaki Ohno, Nobuhisa Mizuki

**Affiliations:** 1 Department of Ophthalmology and Visual Science, Yokohama City University Graduate School of Medicine, Yokohama, Kanagawa 236-0004 Japan; 2 Inflammatory Disease Section, National Human Genome Research Institute, National Institutes of Health, Bethesda, Maryland 20892 USA; 3 Department of Ophthalmology, Hokkaido University Graduate School of Medicine, Sapporo, Hokkaido 060-8638 Japan

## Abstract

Behçet’s disease (BD) is reportedly associated with polymorphisms of the ubiquitin-associated domain containing 2 (*UBAC2*) gene in Turkish, Italian, and Chinese populations. Here we investigated whether *UBAC2* polymorphisms were associated with BD in a Japanese population. Using data from 611 Japanese BD patients and 737 Japanese controls who participated in our previous genome-wide association study, we analyzed the 58 genotyped single-nucleotide polymorphisms (SNPs) in the region 100 kb upstream and downstream of *UBAC2*. We also performed imputation analysis in the region, with 562 imputed SNPs included in the statistical analyses. Association testing revealed that the T allele of rs9517723 in the lncRNA *LOC107984558* was significantly associated with ocular and central nervous system (CNS) lesions and showed the strongest association under the recessive model (TT vs. CT+CC: ocular lesion, *P*c = 0.0099, OR = 1.56; CNS lesion, *P*c = 0.0052, OR = 3.42). Expression analysis revealed that rs9517723 TT homozygotes showed significantly increased *UBAC2* expression (*P* < 0.05). Our findings suggest that enhanced *UBAC2* expression associated with the homozygous risk allele (TT) of rs9517723 could induce overactivation of ubiquitination-related pathway, resulting in the development of ocular and CNS lesions in BD.

## Introduction

Behçet’s disease (BD) is a rare, chronic, systemic, inflammatory disorder characterized by recurrent ocular symptoms, oral and genital ulcers, and skin lesions. In some cases, BD is associated with inflammation throughout the body, for example, in the joints, vascular system, lungs, gastrointestinal tract, central nervous system, and epididymis^1^. BD has been diagnosed worldwide, although it is most commonly found in Mongoloid populations, and rarely in Caucasian populations, and it shows a particularly high prevalence in countries along the ancient Silk Route from Japan to the Middle East and the Mediterranean basin^2^.

Although the etiology of BD remains unclear, several external environmental factors appear to trigger BD in individuals of a particular genetic background. The human leucocyte antigen (HLA) HLA-B*51 is the major susceptibility gene responsible for BD^3–5^. However, HLA-B*51-negative patients can also develop BD, suggesting the involvement of other genetic factors.

BD is also reportedly associated with single-nucleotide polymorphisms (SNPs) of the ubiquitin-associated domain containing 2 (*UBAC2*) gene region on chromosome 13q32.3. Fei *et al*. performed a genome-wide association study (GWAS) using pooled DNA in a Turkish population, and initially reported that BD was potentially associated with a *UBAC2* intronic SNP (rs9513584)^6^. Their further investigation of 14 SNPs in *UBAC2* confirmed the association between *UBAC2* and BD in the Turkish and Italian populations, and revealed that the intronic SNP rs7999348 was the most likely candidate among the genotyped SNPs^7^. Subsequently, Hou *et al*. performed a replication study with 25 SNPs in *UBAC2*, and reported that the promoter SNP rs3825427 showed the most significant association with BD in a Han Chinese population^8^. Overall, these findings suggest that *UBAC2* variants are an important risk factor for BD susceptibility in multiple populations. However, the association between *UBAC2* variants and BD has not yet been assessed in a Japanese population.

The aim of the present study was to investigate whether genetic variants in the *UBAC2* region are associated with BD in a Japanese population. We performed a comprehensive association analysis of SNPs in the *UBAC2* region among Japanese patients with BD.

## Results

### Comprehensive allelic association analysis

**Figure 1:**
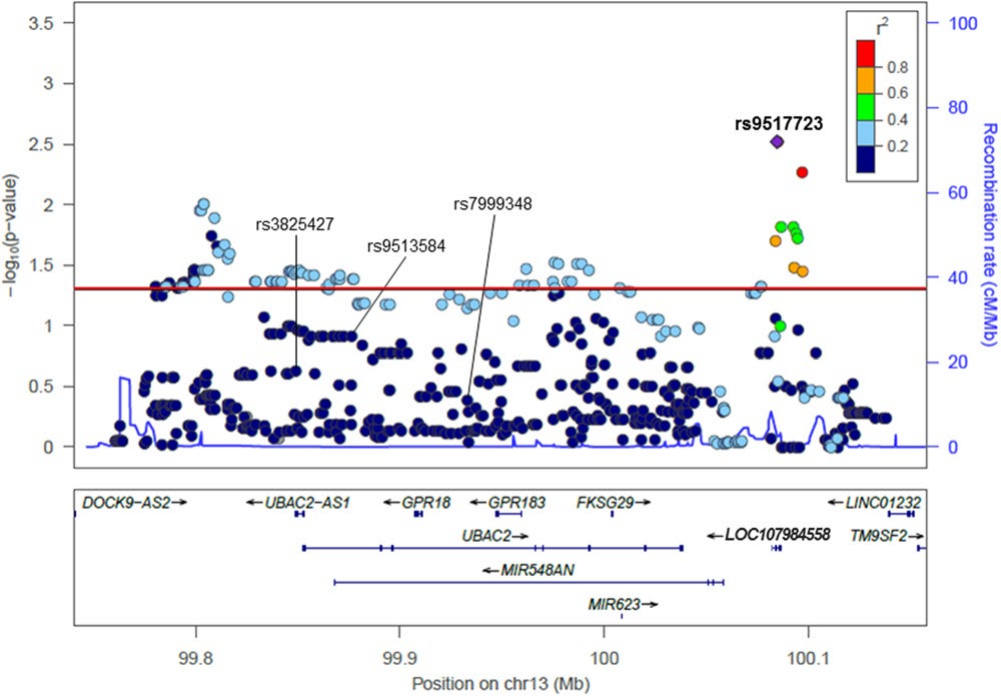
In-depth SNP analysis of the *UBAC2* region. The lead SNP (rs9517723) is depicted as a purple diamond. The color coding of all other SNPs indicates linkage disequilibrium (LD) with the lead SNP: red, r^2^ ≥ 0.8; yellow, 0.6 ≤ r^2^ < 0.8; green, 0.4 ≤ r^2^ < 0.6; cyan, 0.2 ≤ r^2^ < 0.4; blue, r^2^ < 0.2; and gray, r^2^ is unknown. The left y axis represents the −log10 *P* values for allelic association with Behçet’s disease, and the right y axis represents the estimated recombination rate. The horizontal red line indicates the significance level of *P* = 0.05. Gene annotations are shown below the figure. The plot was created using LocusZoom.

Our allelic association analysis in a Japanese population included a total of 620 SNPs (58 genotyped and 562 imputed). Of these SNPs, 100 SNPs showed an association with BD with a *P* value of <0.05 (Fig. 1). The strongest association was observed for rs9517723 located on *LOC107984558*, which showed an increased frequency of its major allele (T) in cases compared to controls (*P* = 0.0024; odds ratio (OR) = 1.27). However, this increase did not reach significance after correcting for multiple testing (*P*c = 0.13) (Table 1). While the SNPs rs3825427, rs9513584 and rs7999348 were previously reported to be associated with BD, we found no such associations in our present population.

**Table 1:** Allelic association results for rs3825427, rs9513584, rs7999348, and rs9517723 in the UBAC2 region.

SNP^a^	Position on Chr. 13 (GRCh37)	Alleles (1 > 2)^b^	Risk Allele	Allele Frequency, %		*P*	*P*c^c^	OR (95% CI)^d^
				Cases (N = 611)	Controls (N = 737)			
rs3825427	99,848,971	C>A	A	38.7	36.4	0.24		1.10 (0.94–1.29)
rs9513584	99,876,281	G>A	G	59.2	56.3	0.12		1.13 (0.97–1.32)
rs7999348	99,932,922	G>A	G	63.0	61.5	0.41		1.07 (0.91–1.25)
rs9517723	100,084,679	T>C	T	63.3	57.5	0.0024	0.13	1.27 (1.09–1.49)

^a^rs9513584, rs7999348, and rs9517723 were genotyped on the GWAS panel while rs3825427 was imputed with the 1000 Genomes reference panel. ^b^1, major allele; 2, minor allele. ^c^ *P*c, corrected *P*; If the *P*c value is greater than 1, it is set to 1. ^d^OR, odds ratio; CI, confidence interval.

### LD analysis

We observed long-range LD across the *UBAC2* gene region. The strongest signal was for rs9517723, which exhibited a strong or moderate LD (r^2^ ≥ 0.2) with many of the other 99 SNPs that showed a significant *P* value of < 0.05 (Fig. 1). However, rs9517723 exhibited very moderate or low LD (r^2^ < 0.2) with many of the remaining 520 SNPs with *P* values of >0.05. Among the 100 SNPs showing a significant *P* value of <0.05, we performed stepwise regression analysis to test the independence of multiple possible associations in the region. Conditioning by rs9517723 eliminated the association of the other 99 SNPs (*P* > 0.05), indicating that rs9517723 could account for most of the association of these SNPs with BD in the Japanese population. On the other hand, rs9517723 was in very moderate or low LD with the previously reported BD-associated SNPs (rs3825427: r^2^ = 0.07; rs9513584: r^2^ = 0.15; rs7999348: r^2^ = 0.10).

### Association between rs9517723 and clinical symptoms of BD

We also analyzed the relationships between rs9517723 and clinical symptoms of BD (Table 2). An allelic association test in the Japanese population revealed that the T allele of rs9517723 was significantly associated with increased risk of ocular and central nervous system (CNS) lesions, with a stronger association with CNS lesions: ocular lesion, *P*c = 0.018, OR = 1.36; CNS lesion, *P*c = 0.0066, OR = 2.78. Table 3 shows the genotypic association results for rs9517723. In both additive and recessive models, we found an association with the disease at *P* < 0.05 among all entire Japanese patients; however, this association did not reach significance after correction (*P*c > 0.05). On the other hand, the T allele of rs9517723 was significantly associated with increased risk of ocular and CNS lesions under the additive and recessive models. The OR was stronger among patients with CNS lesions, and the associations with both ocular and CNS lesions were stronger in the recessive model (ocular lesion: *P*c = 0.0099, OR = 1.56; CNS lesion: *P*c = 0.0052, OR = 3.42) than in the additive model (ocular lesion, *P*c = 0.023, OR = 1.34; CNS lesion, *P*c = 0.0078, OR = 2.94). Moreover, the T allele of rs9517723 was associated with 1.33-fold to 1.42-fold increased risks of oral ulcer, skin lesion, genital ulcer, and arthritis; however, these risks were not significant (*P* < 0.05, *P*c > 0.05).

**Table 2:** Allelic association results between rs9517723 and clinical symptoms of Behçet’s disease.

Phenotype		N	Risk Allele (T) Freq., %	*P*	*P*c^a^	OR (95% CI)^b^
Controls		737	57.9			
Cases	ALL	611	63.0	0.0024	0.13	1.27 (1.09–1.49)
	Oral ulcer	589	63.4	0.0037	0.19	1.26 (1.08–1.48)
	Skin lesion	510	62.7	0.015	0.76	1.23 (1.04–1.44)
	Ocular lesion	491	65.1	0.00034	0.018	1.36 (1.15–1.60)
	Genital ulcer	372	64.5	0.0026	0.13	1.32 (1.10–1.59)
	Arthritis	229	62.7	0.069		1.22 (0.98–1.52)
	Epididymitis	38	61.8	0.49		1.18 (0.73–1.90)
	GI lesion^c^	100	61.5	0.33		1.16 (0.86–1.57)
	Vascular lesion	29	65.5	0.25		1.38 (0.80–2.40)
	CNS lesion^d^	41	79.3	0.00013	0.0066	2.78 (1.62–4.80)

^a^ *P*c, corrected *P*. ^b^OR, odds ratio; CI, confidence interval. ^c^GI, gastrointestinal. ^d^CNS, central nervous system.

**Table 3:** Genotypic association results between rs9517723 and clinical symptoms of Behçet’s disease.

Phenotype		N	Genotype ((T/T)/(C/T)/(C/C)) Frequency %	Genetic Models								
				Additive (T/T vs. C/T vs. C/C)			Dominant (T/T+C/T vs. C/C)			Recessive (T/T vs. C/T+C/C)		
				*P*	*P*c^a^	OR (95% CI)^b^	*P*	*P*c^a^	OR (95% CI)^b^	*P*	*P*c^a^	OR (95%CI)^b^
Controls		737	33.6/48.4/17.9									
Cases	ALL	611	41.4/44.1/14.5	0.0028	0.14	1.27 (1.09–1.48)	0.083		1.30 (0.97–1.74)	0.0025	0.13	1.41 (1.13–1.76)
	Oral ulcer	589	41.6/43.6/14.8	0.0044	0.23	1.25 (1.07–1.46)	0.13		1.26 (0.94–1.69)	0.0029	0.15	1.40 (1.12–1.76)
	Skin lesion	510	40.2/45.1/14.7	0.016	0.81	1.22 (1.04–1.44)	0.14		1.27 (0.93–1.72)	0.018	0.94	1.33 (1.05–1.67)
	Ocular lesion	491	44.2/41.8/14.1	0.00045	0.023	1.34 (1.14–1.58)	0.074		1.33 (0.97–1.83)	0.00019	0.0099	1.56 (1.23–1.97)
	Genital ulcer	372	41.9/45.2/12.9	0.0027	0.14	1.32 (1.10–1.58)	0.033	1.00	1.47 (1.03–2.10)	0.0068	0.35	1.42 (1.10–1.84)
	Arthritis	229	41.0/43.2/15.7	0.072		1.21 (0.98–1.50)	0.45		1.17 (0.78–1.75)	0.041	1.00	1.37 (1.01–1.86)
	Epididymitis	38	34.2/55.3/10.5	0.49		1.16 (0.71–1.90)	0.24		1.85 (0.65–5.32)	0.94		1.03 (0.52–2.04)
	GI lesion^c^	100	37.0/49.0/14.0	0.33		1.15 (0.85–1.57)	0.33		1.34 (0.74–2.43)	0.51		1.16 (0.75–1.79)
	Vascular lesion	29	34.5/62.1/3.4	0.25		1.38 (0.77–2.47)	0.044	1.00	6.11 (0.82–45.3)	0.93		1.04 (0.48–2.27)
	CNS lesion^d^	41	63.4/31.7/4.9	0.00015	0.0078	2.94 (1.65–5.22)	0.032	1.00	4.25 (1.01–17.84)	0.00010	0.0052	3.42 (1.78–6.57)

^a^ *P*c, corrected *P*; If the *P*c value is greater than 1, it is set to 1. ^b^OR, odds ratio; CI, confidence interval. ^c^GI, gastrointestinal. ^d^CNS, central nervous system.

### Expression analysis

**Figure 2:**
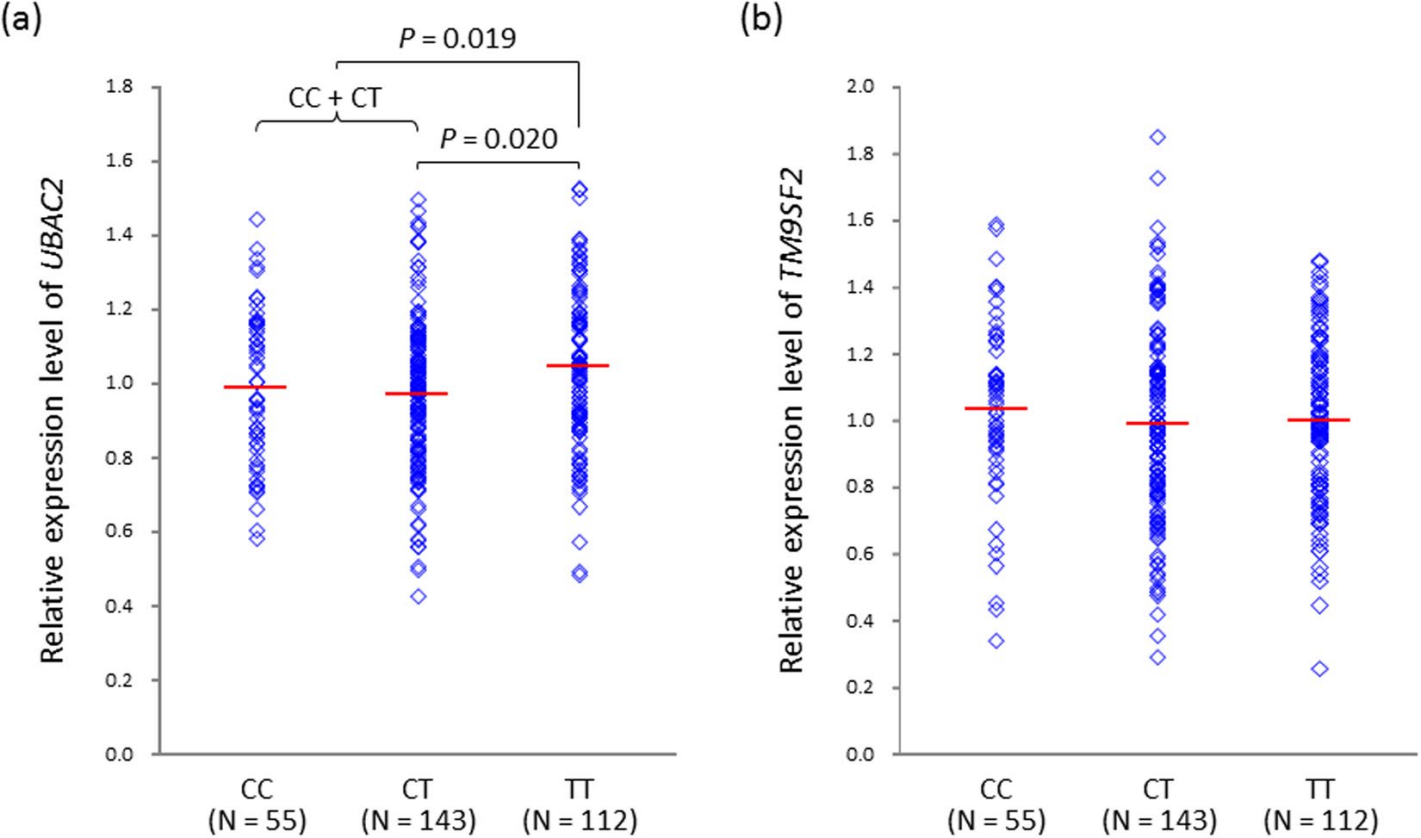
Expression analysis of *UBAC2* (**a**) and *TM9SF2* (**b**) stratified by rs9517723 genotype.

The SNP rs9517723 is located on the first exon of *LOC107984558*, which encodes a long non-coding RNA (lncRNA) and is located between the protein coding genes, *UBAC2* (43 kb downstream) and *TM9SF2* (68 kb upstream) (Fig. 1). Through a variety of mechanisms, lncRNA can regulate gene expression in cis or in trans^9, 10^. Thus, we investigated whether rs9517723 affected the expression level of *UBAC2* and/or *TM9SF2*. *UBAC2* expression was significantly increased in the rs9517723 TT homozygotes (TT vs. CT, *P* = 0.020; TT vs. CC+CT, *P* = 0.019) (Fig. 2a). The rs9517723 genotype was not associated with *TM9SF2* expression level (Fig. 2b).

## Discussion

In our present study, we aimed to assess whether genetic variants in the *UBAC2* region affected BD development in a Japanese population. We performed comprehensive association analysis of SNPs in the region 100 kb upstream and 100 kb downstream of *UBAC2* among Japanese patients with BD. This study is the first comprehensive investigation of the *UBAC2* region for association with BD. We found that SNP rs9517723 in the lncRNA *LOC107984558* was recessively associated with the risks of ocular and CNS lesions (showing a stronger association with CNS lesions) but not with BD itself in the Japanese population. This suggests that rs9517723 contributes to the development of ocular and CNS lesions, especially CNS lesions, with a recessive effect.

*UBAC2* encodes an ubiquitination-related structural domain that is implicated in ubiquitination and proteasomal degradation. BD is reportedly associated with other ubiquitination-related genes, including ubiquitin associated and SH3 domain containing B (*UBASH3B*)^6^, small ubiquitin-like modifier 4 (*SUMO4*)^11–13^, and ubiquitin-conjugating enzyme E2Q family-like 1 (*UBE2QL1*)^14^, suggesting that the ubiquitination-related pathway may be involved in BD pathophysiology. In our present study, we found that the homozygous risk allele (TT) of the lncRNA *LOC107984558* rs9517723 was significantly correlated with enhanced *UBAC2* expression. Sawalha *et al*. also showed that *UBAC2* expression was significantly increased in the homozygous risk allele of a BD-associated SNP (the GG genotype of rs7999348)^7^. While their study did not assess associations between the allele and clinical symptoms of BD^7^, their findings and our present results suggest that the enhanced *UBAC2* expression associated with these homozygous risk alleles could induce overactivation of the ubiquitination-related pathway, resulting in the development of ocular and CNS lesions or of BD itself. On the other hand, Hou *et al*. showed that the risk allele of their identified BD-associated SNP (the T allele of rs3825427) led to downregulated *UBAC2* expression^8^, suggesting that decreased *UBAC2* expression also contributes to BD risk. These contradictory findings indicate a need for further functional studies to clarify how *UBAC2* contributes to BD pathophysiology.

LncRNAs are defined as non-coding RNA transcripts of more than 200 nucleotides in length. Recent evidence shows that lncRNAs play key functional roles in diverse biological processes, including chromatin remodeling, transcriptional, posttranscriptional, and epigenetic regulation^9, 10, 15^. They also contribute to the pathophysiology of various diseases, including cancers and neurological, autoimmune, and ocular diseases^15–19^. Moreover, genetic variants in lncRNAs can modulate the structure and expression of localized lncRNAs, leading to functional alterations of their interacting partners^20–22^. The available data regarding lncRNAs may support our present findings that rs9517723 in the lncRNA *LOC107984558* leads to enhanced *UBAC2* expression, contributing to BD pathophysiology. In general, lncRNA expression is more tissue-specific than the expression of protein-coding genes, and most lncRNAs are highly expressed in the CNS with low expression in other tissues^23–25^. This characteristic expression pattern of lncRNAs may explain why rs9517723 in the lncRNA *LOC107984558* is more strongly associated with CNS lesions than with other BD symptoms. However, we did not assess the expression pattern of *LOC107984558* in our current study, nor did we find public databases containing *LOC107984558* expression data.

In conclusion, our present findings indicate that rs9517723 in the lncRNA *LOC107984558* was significantly associated with increased risks of ocular and CNS lesions within a Japanese population. Our results further suggest that rs9517723 is associated with enhanced *UBAC2* expression, which contributes to the development of those lesions. Further validation studies in other ethnic populations are needed to confirm these findings. Additionally, further expression analyses using RNA isolated from cells of ocular and CNS lesions in patients with BD are needed to more clearly elucidate the effect of 9517723 on *UBAC2* expression. In the future, rs9517723 may serve as a useful genetic marker for BD diagnosis, especially in cases with CNS lesions.

## Methods

### Subjects

Our previous GWAS enrolled 611 unrelated Japanese individuals with BD, and 737 unrelated Japanese controls^26^. Here we used genotype data from that study, specifically for the 58 SNPs found from 100 kb upstream to 100 kb downstream of the *UBAC2*. All 58 SNPs satisfied the following quality control criteria: a call rate >98%, Hardy-Weinberg equilibrium (HWE) *P* > 0.001, and minor allele frequency >1%. The Japanese patients were diagnosed with BD according to the standard criteria^27^ proposed by the Japan Behçet’s Disease Research Committee. All control participants were healthy volunteers, who were unrelated to each other or to the patients. All participants gave their written informed consent. The study methodology adhered to the tenets of the Declaration of Helsinki, and was approved by the Ethics Committee of Yokohama City University School of Medicine.

### Imputation analysis of the *UBAC2* gene region

To evaluate potential associations with un-genotyped SNPs within the region encompassing the *UBAC2* gene, we performed imputation analysis. The genotypes of our Japanese GWAS set were imputed based on the 58 genotyped SNPs using MACH v1.0 (http://www.sph.umich.edu/csg/abecasis/MACH/index.html)^28, 29^. The reference panel comprised the 1000 Genomes Phase 3 datasets of 315 East Asian samples, which included a set of Japanese samples from Tokyo (JPT, N = 104), Han Chinese samples from Beijing (CHB, N = 103), and Southern Han Chinese samples (CHS, N = 108)) (http://www.1000genomes.org/)^30^. All imputed SNPs were filtered with the following quality control settings: HWE *P* > 0.001, minor allele frequency >1%, and a squared correlation between imputed and true genotypes (Rsq) of >0.90. A total of 562 imputed SNPs were included in further analysis.

### Expression analysis

From our genome-wide expression (GWE) dataset, we obtained expression data for the *UBAC2* and transmembrane 9 superfamily member 2 (*TM9SF2*) genes from 313 Japanese healthy volunteers (Meguro *et al*., unpublished data). The GWE analysis was performed using the Illumina HumanHT-12 v4 Expression BeadChip Kit. First, whole blood was collected from subjects in PAXgene Blood RNA tubes (Becton Dickinson, Heidelberg, Germany), and total RNA was extracted from whole blood using the PAXgene Blood RNA Kit (Qiagen) following the manufacturers’ protocols. Next, the total RNA samples were processed using the TargetAmp-Nano Labeling Kit for the Illumina Expression BeadChip (Epicentre, Wisconsin, USA) and hybridized to the BeadChips following the manufacturers’ protocols.

### Statistical analysis

We performed allelic and genotypic association analyses and stepwise regression analyses, and calculated linkage disequilibrium (LD) using SNP & Variation Suite software version 8.6.0 (Golden Helix, Inc., Bozeman, MT, USA). A correlation/trend test was used to assess differences in allele and genotype frequencies between cases and controls. We generated a regional association plot for the *UBAC2* region using LocusZoom (http://csg.sph.umich.edu/locuszoom/)^31^. Tagging SNPs were selected from the genotype data of the Japanese GWAS set (611 BD patients and 737 controls) with an r^2^ threshold of 0.80, using PLINK version 1.07 (http://pngu.mgh.harvard.edu/purcell/plink/)^32^. This identified 52 tagging SNPs, capturing all 620 SNPs (58 genotyped and 562 imputed SNPs) within the *UBAC2* region. The obtained *P* values were corrected for multiple testing using Bonferroni correction based on the number of tagging SNPs. A corrected *P* (*P*c) value of < 0.05 was considered significant. Differences in the expression levels of *UBAC2* and *TM9SF2* were analyzed using the Mann-Whitney U test.
